# Perceptions of Intentionality for Goal-Related Action: Behavioral Description Matters

**DOI:** 10.1371/journal.pone.0119841

**Published:** 2015-03-17

**Authors:** Andrew E. Monroe, Glenn D. Reeder, Lauren James

**Affiliations:** 1 Department of Psychology, Florida State University, Tallahassee, Florida, United States of America; 2 Department of Psychology, Illinois State University, Normal, Illinois, United States of America; 3 Department of Psychology, DePaul University, Chicago, Illinois, United States of America; University of Amsterdam, NETHERLANDS

## Abstract

Perceptions of intentionality critically guide everyday social interactions, though the literature provides diverging portraits of how such judgments are made. One view suggests that people have an "intentionality bias," predisposing them toward labeling behaviors as intentional. A second view focuses on a more complex pattern of reasoning whereby judgments of intentionality are shaped by information about social context and mental states. Drawing on the theory of action-identification, we attempt to integrate these two perspectives. We propose that people parse intentionality into two categories: judgments about concrete, low-level behaviors and judgments about relatively more abstract, high-level behaviors. Evidence from five studies supports this distinction. Low-level behaviors were perceived as intentional regardless of mental state information, supporting the “intentionality bias” view. In contrast, judgments about the intentionality of high-level behaviors varied depending on social context and mental states, supporting the systematic view of intentionality.

## Introduction

Inferences of intentionality crucially guide everyday social, moral, and legal life. Intentionality influences the types of explanations people offer for behavior [1–2], and intentionality shapes social and moral evaluations of others [3–6]. Even the experience of pain seems to be susceptible to perceptions of intent [7].

Studies on perceived intentionality, however, often stress one of two themes [4, 8–11]. On the one hand, some research suggests that judgments of intentionality are simplistic and subject to various biases including the valence of the behavior (e.g., [9, 12–13]) as well as a more general “intentionality bias” [14–16], whereby “we view *everything anyone ever does as intentional*” ([15], p. 772 italics Rosset’s). On this account people interpret even prototypically accidental behaviors (e.g., “She broke the vase.”) as intentional by default and judgments of unintentionality are only achieved by overriding the default judgment.

On the other hand, a separate line of research argues that judgments of intentionality are systematic and thoughtful [1, 17–21]. In particular, people sometimes carefully consider information about social context and motive when judging intentionality. For instance, consider a scenario where an agent harmed an innocent victim by delivering electric shock [20]. If an experimenter coerced the agent into delivering the shocks, or if the agent appeared to want to help the victim, people tended to view the harmful behavior as unintentional. In contrast, if coercion was absent or if the agent had hurtful motives people tended to rate the behavior as intentional. In addition, the folk concept of intentionality is comprised of five distinct criteria—including an agent’s beliefs and desires—that people consider when making intentionality judgments [19]. Thus, this work implies that judgments of intentionality depend on the social context in which behavior occurs and on consideration of an agent’s mental states.

### Action Identification and Intentionality Judgments

We propose that these two themes in the literature can be integrated to some extent by examining the different levels at which behavior can be conceptualized. Following from action identification theory [22–23], we propose that people categorize and explain behavior either by referring to its low-level, concrete properties (e.g., raising one’s hand, signing a check) or by referencing a behavior’s high-level, abstract goals (e.g., challenging a speaker’s point of view, helping underprivileged children). These levels of action identification have different cognitive [24] and neural representations [25]; they correspond to different phases of action specification (e.g, motor vs. distal intentions, [26]), and they differentially rely on mental state ascriptions [27].

Using action identification as a lens, we propose that work on the intentionality bias [14–15] tends to focus on people’s judgments of intentionality for specific, concrete behaviors (e.g., he pressed the delete button). In contrast, work demonstrating the contextual sensitivity of perceived intentionality [20] focuses on the abstract effects of behavior (e.g., harming a victim).

In this paper, we attempt to integrate these two literatures by distinguishing between judgments of intentionality for low-level, concrete behavior descriptions and judgments of intentionality for high-level, abstract behavior descriptions.

Work on adult social cognition shows that people quickly and easily judge the intentionality of concrete acts [28–29], often relying on low-level perceptual cues [30–31] or robust social scripts [32]. Because of this, we predict that judgments of intentionality for concrete behavior descriptions should be consistent with research by Rosset and colleagues [14–15]. For example, judging whether an agent “picked up the pen” intentionally should not require information about social context or agents’ mental states. Instead, these judgments should be strongly informed by low-level cues—such as the near-term, goal-directedness of the agent’s movements (aiming to grasp the pen) or the verbs used to describe behavior. The direct path of the agent’s hand to the pen or the linguistic heuristics associated with the verb phrase “picked up” clearly marks the behavior as intentional. Judgments of this sort can be made without knowing the person’s ultimate goals (e.g., wanting to write checks to combat world hunger). Indeed, infants who are not yet able to conceptualize agents’ mental states easily recognize such behavior as intentional [33–34].

In contrast, judgments of intentionality for abstract behaviors are more complex and rely, in part, on information about social context, and on what that context implies about the agents’ mental states [4, 20, 35]. Unlike judgments of concretely described behaviors, where people may assume that the opposite of acting intentionally is acting accidentally, judgments of abstractly described behavior are likely more nuanced. In this latter case, judging intentionality may require that an agent has a specific, behavior-congruent, desire on his mind when he acts [20]. Thus, while all of the low-level, concrete behaviors leading up to an a high-level outcome might be intentional (e.g., he picked up the gun, aimed it, and pulled the trigger), if an agent lacks a specific, behavior-congruent, desire (e.g., wanting to kill his friend) people may be reluctant to attribute responsibility and intentionality to the agent [36].

Similarly, Guglielmo and Malle [4] show that providing people with information about an agent’s mental states (desire or regret) shapes intentionality judgments for high-level behaviors. In their studies they described a situation in which a CEO made a decision that had the side-effect of harming the environment. In one condition, a company CEO was described as regretfully harming the environment (“It would be unfortunate if the environment got harmed. But my primary concern is to increase profits. Let’s start the new program” p. 1639). In a second condition, however, the CEO declared, “I don’t care at all about harming the environment.” Results showed that the CEO’s expression of regret reduced participants’ willingness to say that the CEO intentionally harmed the environment (40% for the ‘regretful’ CEO and 82% for the ‘uncaring’ CEO). In short, the CEO’s motivation toward the environment was crucial in determining whether people saw the harm as intentional.

Often times the social context provides cues about an agent’s mental states. In particular, social context can carry information about an agent’s beliefs and motives, which in turn, shape inferences of intentionality, at least for high-level behaviors [20–21]. For instance, Monroe and Reeder [20] told participants about a wealthy businessman who donated $10 million dollars to underprivileged children. The social context was manipulated such that the donation took place under either low psychological coercion (no external threat) or high psychological coercion (death threats from terrorists). Participants were asked to judge if the businessman intentionally “helped the children”. The presence of high coercion reduced judgments of intentionality (compared to the low coercion condition), and this effect was mediated by perceptions of motive. The mediation suggests that judgments of intentionality followed a “motive-matching” tendency. In the high coercion condition, perceivers assumed the businessman was motivated to save his own life. Because this self-serving motive did not match the charitable behavior he produced (i.e., helping underprivileged children), people judged that his helping the children was unintentional. By contrast, the businessman in the low coercion condition was seen as motivated to help the children—a motive that matched his behavior. Consequently, judgments of intentionality were relatively high in this condition.

Thus, it appears that perceivers rely on the social context to disambiguate the meaning of abstractly construed behavior. In the current studies we therefore predicted that judgments of intentionality for such behavior would be sensitive to manipulations of the social context. But what about concretely construed behavior? Our analysis suggests that concrete behaviors (e.g., reaching toward a pen) are processed in terms of low-level perceptual cues (e.g., directedness of an agent’s reaching motion) [37–38]. Consequently, the interpretation of concrete behaviors can proceed without drawing upon the broader social context for added meaning. It therefore follows that such intentionality judgments should be relatively robust against manipulations of social context.

### Comparing the Present Studies to Past Research

At first glance, two previous investigations appear to offer conflicting evidence on our proposal. In line with our view, Molden [39] reported that low-level behavior descriptions (e.g., Ben stole some bread from a bakery) were judged as more intentional than high-level behavior descriptions (e.g., Tony received many complaints about his work). On the other hand, Kozak and her colleagues [27] reported that low-level action identification (e.g., pulling fruit from a tree vs. getting something to eat) was negatively associated with their measure of perceived intentionality. In our view, neither of these lines of investigation offers a fair test of our predictions. First, Molden [39] deliberately selected high-level behavioral descriptions (what he calls “outcomes”) that were not directly related to the target person’s low-level actions (what he calls “behavior”) and, thus, implied low intentionality (“By definition, the outcomes described involved less choice by the actor and therefore should be rated as less intentional than the behaviors.” p. 42). It is clear, therefore, that Molden did not intend his research to be a test of the idea that low-level behavior descriptions are typically seen as more intentional than high-level behavior descriptions. Second, the relevant evidence from the Kozak et al. [27] studies is correlational and did not directly compare low- and high-level descriptions of behavior in terms of intentionality. In addition, their measure of intentionality never asked directly about the intentionality of a specific behavioral event, but rather inquired about the capabilities of an agent (e.g., is this person capable of planning, purposeful action, and having goals?).

The current studies address these limitations by asking all participants within a given condition to read the same scenario and then answer questions about the intentionality of both concrete behavioral descriptions (e.g., calling an accountant; writing a check) and closely related abstract behavior descriptions (e.g., helping underprivileged children; doing a good deed). Accordingly, the relatively low-level behaviors appeared instrumental in producing the more abstractly described high-level behaviors. Of equal importance, the current research investigates how people glean mental state information from the social context and use this information to judge the intentionality of low-level and high-level behaviors.

Two main hypotheses guided the research. First, because judgments of intentionality for low-level behaviors are guided by simple heuristic cues (e.g., intention-signaling verbs), we predicted that people would judge low-level behavior descriptions as more intentional than high-level behavior descriptions. Second, we expected that manipulations of social context (e.g., coercion, the interaction history between agents) would have greater impact on perceived intentionality for high-level behavior descriptions, as opposed to low-level behavior descriptions. In our model, the social context provides information about an agent’s mental states which motivate high-level goals [21]. Accordingly, when the agent’s mental states appear to match the obtained high-level goal, perceptions of intentionality are increased [20].

In Study 1 participants judged the intentionality of various pro-social low and high-level behavior descriptions, in the context of either low coercion (free choice) or high coercion (terrorist threat). Study 2 examined judgments of intentionality for anti-social (aggressive) behavior, which is a focus of the majority of previous research. Additionally, Study 2 employed a more subtle manipulation of social context (the target person’s aggression was in response to friend who either helped or refused to help the target person). Study 3 tested an alternative interpretation of our findings whereby low-level behaviors are perceived as more intentional than high-level behaviors because low-level behaviors have comparatively fewer intermediary steps between forming an intention and completing an act. Finally, Studies 4 and 5 (see General Discussion) addressed two additional alternative explanations. Study 4 tested whether the findings occur because low-level behavioral descriptions (signing a check) seem morally neutral whereas high-level descriptions (helping needy children) seem morally valenced. Study 5 examined the possibility that our findings were driven by an artifact related to the wording of our questions (i.e., asking participants if behaviors were *intentional*, rather than *accidental*).

## Study 1

Study 1 was designed to explore the general idea that judgments of intentionality for low-level and high-level behaviors are distinct from one another. Specifically, this study tested the predictions that (a) low-level behavior descriptions would be perceived as more intentional than high-level behavior descriptions, (b) judgments about high-level (but not low-level) behavior descriptions would be highly influenced by social context, and (c) inferences about mental states (motive) would mediate the influence of social context on intentionality judgments for high-level behavior descriptions.

### Methods

#### Ethics Statement

The Illinois State University, Brown University, and Florida State University Institutional Review Boards approved the ethics of all of the following studies. All participants were 18 years of age or older and provided written informed consent (Studies 1 and 2) or indicated their consent using an online form (Studies 3–5).

#### Participants and Procedure

Participants were recruited from a public Midwestern University. Fifty-six undergraduate students (54 female, mean age 19.4, *SD* = 2.45) participated in the experiment in exchange for course credit.

After arriving at the experiment, participants read and signed the informed consent form, and were randomly assigned to read one of two vignettes about a wealthy businessman who made a large donation ($10 million) to a local school system serving underprivileged children (adapted from 20). The vignettes manipulated the amount of coercive pressure acting on the businessman to donate the money to the school (low coercion or high coercion). After reading the vignette, participants provided a series of ratings of intentionality and the businessman’s motives.

#### Coercion Manipulation

In the low coercion condition the businessman was described as reflecting on the fact that he had not donated money in several years and then deciding to donate to a school for the underprivileged:

“A businessman became rich and is now worth about 100 million dollars. But in the last few years, he has not given any money at all to charitable causes. The businessman went to bed early on Sunday night.

Monday morning, he called his accountant and instructed him to arrange it so that he can give money to a local school system that serves mainly underprivileged kids. The businessman then met with school officials, wrote a 10 million dollar check, and handed it to a school official.”

The high coercion condition was identical to the low coercion condition except that the first paragraph was altered to include a description of the businessman being threatened by a group called the “Radical Socialists” who wanted him to donate to a school for underprivileged children:

“A businessman became rich and is now worth about 100 million dollars. But in the last few years, he has not given any money at all to charitable causes. Sunday night members of a group calling itself the "Radical Socialists" broke into his home at night and tied him up. They told him that unless he donated 10 million to a local school that serves poor children, they would return in one week and kill him. They said if he went to the police, they would still find a way to kill him. The group then untied him and left his home.”

#### Ratings of Low and High-Level Behavior Descriptions and Motives

After reading the vignette, participants were asked to evaluate the intentionality of three *low-level behavior descriptions* (“Did the businessman intentionally call his accountant”; “…meet with school officials”; “…write a $10 million check”) and three *high-level behavior descriptions* (“Did the businessman intentionally help the children”; “…give money to a charitable cause”; “…do a good deed”), using a 1 (Not at all) – 7 (Completely) Likert scale. In all of the studies in this article, the order of the low-level and high-level behavior questions was counterbalanced across forms. Because counterbalancing produced no significant effects, the results were collapsed across orders.

Additionally, participants were asked to rate the importance of both altruistic motives (wanting to be altruistic; wanting to help underprivileged children) and self-serving motives (wanting to protect himself; wanting to stay alive) for causing the businessman’s behavior on a 1 (Most definitely not) – 7 (Most definitely) Likert scale. At the end of the study participants indicated their age and gender and were thanked for their participation and debriefed.

### Results

#### Data Reduction

The low-level and high-level behavior descriptions demonstrated high internal consistency (α = .90 and α = .86, respectively). Further, the low-level and high-level behavior items were pretested on a separate group of participants for concreteness (*n* = 37). A paired-sample t-test showed that people viewed the three low-level items to be more concrete (*M* = 3.82 *SD* = 1.67) than the three high-level items (*M* = 4.59 *SD* = 1.54), *t*(36) = -3.12, *p* = .004 (Scale: 0 = very concrete; 6 very abstract). Given the high internal consistency and clear differentiation between the items, we combined them into “low-level intent” and “high-level intent” composite measures for all future analyses.

Similarly, the individual altruistic and self-serving motive items were highly correlated (*r* = .63 and *r* = .91, respectively) and we combined each into composite measures. These composite measures of altruistic and self-serving motives were significantly negatively correlated *r*(55) = -.75, *p* < .001, so we computed an overall measure of subjects’ perceptions of the agent’s motives by subtracting the two composites (See S1 Table for descriptive statistics for all individual intentionality and motive items).

#### Effects of Coercion on Perception of Intentionality for Low vs. High-Level Behavior Descriptions

**Fig 1:**
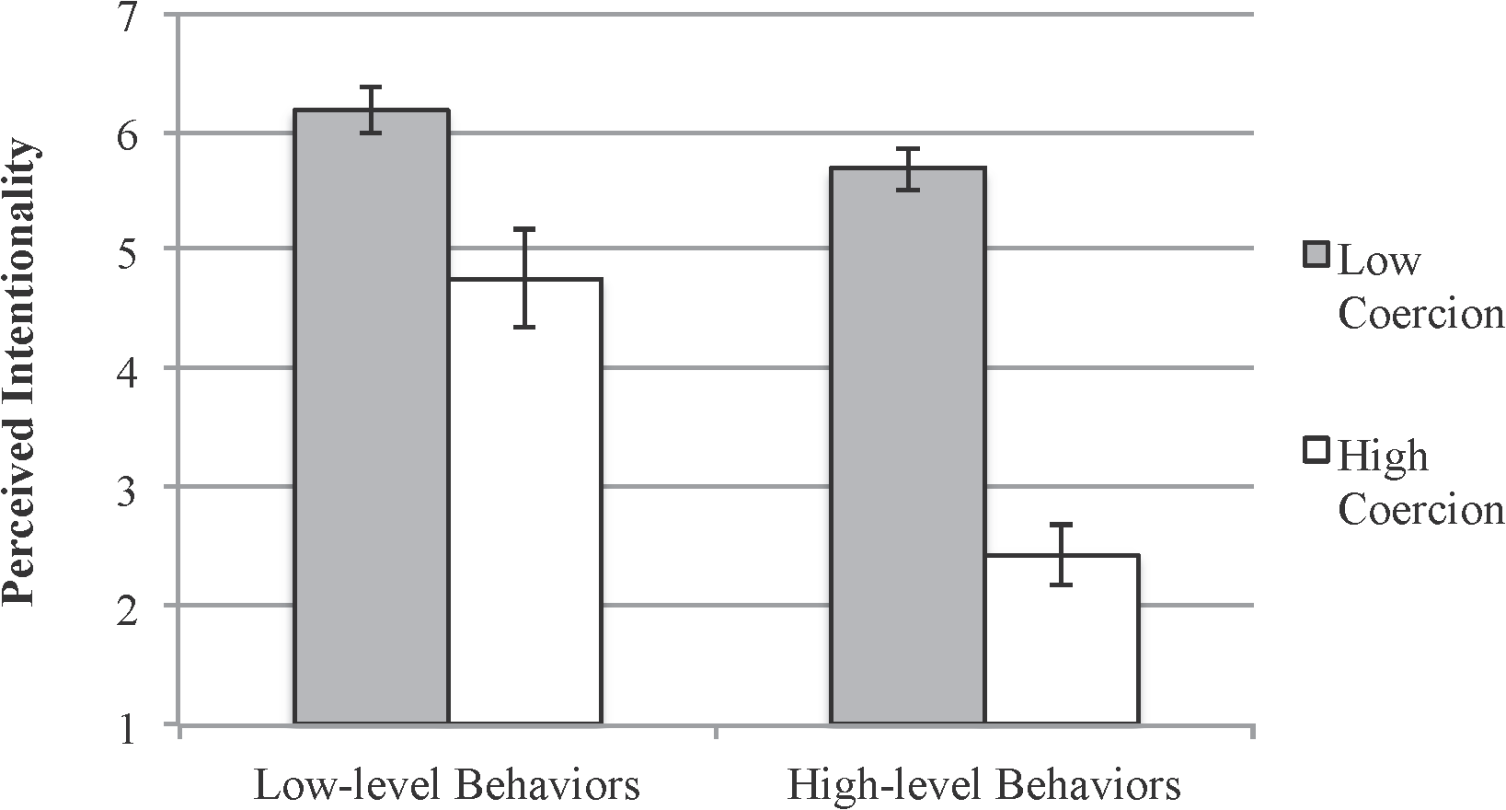
Mean judgments of intentionality for low-level and high-level behavior descriptions by coercion condition. Error bars = ±1 SE.

Perceptions of intentionality are displayed in Fig. 1. We tested our predictions with a 2 (Coercion: low vs. high) x 2 (Type of Event: low-level vs. high-level behavior) mixed model ANOVA, with repeated measures on the second factor. Our first prediction was that low-level behavior descriptions would be perceived as more intentional than high-level behavior descriptions. This prediction received strong support, (*M* = 5.47, *SD* = 1.83 vs. *M* = 4.06, *SD* = 2.00, respectively), *F*(1,54) = 27.9, *p* < .001, *d* = .74. Additionally, there was a significant main effect of coercion on perceptions of intentionality. Replicating previous work (Monroe & Reeder, 2011), participants rated the businessman’s helping behavior as less intentional when he was under strong coercion to donate (*M* = 3.60 *SD* = 1.77), than when coercion was low (*M* = 5.93 *SD* = 0.99), *F*(1,54) = 66.8, *p* < .001, *d* = 1.7.

Our second prediction was that intentionality judgments for high-level behavior descriptions would be sensitive to social context information, whereas intentionality judgments for low-level behavior descriptions would be relatively insensitive to social context information. A significant coercion by event interaction supported this prediction, *F*(1,54) = 11.6, *p* = .001, *d* = 1.29. *High-level behaviors* produced under low coercion were seen as intentional (M = 5.68 *SD* = .92), whereas high-level behaviors produced under high coercion were perceived as unintentional (M = 2.44 *SD* = 1.38), *F*(1,54) = 107.0, *p* < .001, *d* = 2.76. Thus, intentionality judgments of high-level behaviors were highly dependent upon the social context. By contrast, *low-level behaviors* were largely viewed as intentional. Nevertheless, the difference in intentionality between the low coercion (M = 6.18 *SD* = 1.07) and high coercion (M = 4.76 *SD* = 2.16) conditions did attain statistical significance in the case of low-level behavior descriptions, *F*(1,54) = 9.68, *p* = .003, *d* = .83.

Finally, we predicted that perceived motives would mediate the effect of coercion on perceived intentionality for high-level behavior descriptions. Separate regressions indicated that the coercion condition significantly predicted both intentionality for high-level behavior descriptions (β = -.82, *p* < .001) and perceived motives, β = -.88, *p* < .001. When the coercion condition was entered simultaneously with perceived motives, the relationship between condition and *high-level behaviors* diminished (β = -.30, *p* = .05); however, consistent with our motive-matching model, perceived motives remained a highly significant predictor of intentionality (β = .59, *p* < .001). Sobel’s test indicated that the effect of coercion condition was significantly reduced by perceived motives, *z* = -3.58, *p* < .001.

### Study 1 Discussion

Study 1 showed initial support for our hypothesis that judgments of intentionality are distinct depending on whether behaviors are described in terms of their low-level, concrete properties (e.g., picking up a pen) versus their high-level, abstract outcomes (e.g., helping children). These data clarify the role of social and mental state information in judging intentionality. Social and mental state information (e.g., coercion and motive) play an important role in shaping judgments of intentionality for high-level behaviors. By contrast, judgments of intentionality for low-level behavior descriptions were relatively robust. People viewed low-level behaviors as intentional regardless of social information, and they judged low-level behaviors to be more intentional, in general, compared to high-level behaviors.

However, there are two important limitations to the study. First, the study examined judgments of intentionality for pro-social behavior. By contrast, most of the intentionality literature (e.g., [4, 9, 11, 15]) has focused on judgments of intentionality for negative behaviors (e.g., breaking a vase, harming the environment). Thus, if judgments of intentionality for positively and negatively valenced behaviors are fundamentally different, data from Study 1 cannot speak to this debate. Second, a critic could argue that our manipulation of social context via coercion was overly blunt and, therefore, participants could not ignore the effects of the social context on the agent (though arguments to the contrary abound in social psychology, e.g., [40–41]). This begs the question of whether our results would replicate with a less heavy-handed, everyday manipulation of social context.

## Study 2

We addressed each of these limitations in Study 2. First, to address the limitation of behavior valence, we sought to replicate our pattern of results for an aggressive, rather than pro-social, behavior. Second, in Study 2 we used an everyday scenario (a refused request for a favor) as our manipulation of social context, addressing the concern about the bluntness of our manipulation and creating a more stringent test of our hypothesis. As in Study 1, we predicted that low-level behavior descriptions would be judged as more intentional than high-level behavior descriptions. We also predicted that people’s perceptions of the agent’s motives—as manipulated by the social context—would critically influence judgments of intentionality for high-level, but not low-level, behavior descriptions.

### Methods

#### Participants

Subjects were recruited from a public Mid-Western University. In total, 96 subjects participated in the experiment in exchange for course credit. The majority of the sample was female (*n* = 83) with an average age of 20.1 years (*SD* = 2.7).

#### Procedure and Materials

Subjects participated in small groups of 5–15 people. Participants were presented with a written informed consent form. After signing the form, participants were randomly assigned to read one of two short vignettes. Each vignette described a character named Harry who had a difficult upcoming exam and asked his friend Tom for help studying. In the *positive motive condition* Tom agreed to help, and as a result, Harry felt positively about taking the exam (e.g., “Harry took the exam and felt very confident. He was able to answer all the questions with certainty and was sure he would receive an A.”). In the *revenge motive condition*, however, Tom refused to help, and as a result, Harry felt upset about taking the exam (e.g., “Harry took the exam and felt very upset with how it went. He guessed on most of the questions and was sure he failed the exam.”).

After reading one of the two the vignettes, all participants watched the same short video depicting an aggressive interaction between Harry and Tom as the two played a “hand-slap game.” In this game, Tom was shown placing the palms of his hands facing downwards over the upturned palms of Harry. Harry then tried to slap Tom’s hands before Tom could move them out of the way. Although slapping hands was the aim of the game, Harry succeeded in slapping Tom with a surprising amount of force. Thus, Harry’s behavior might best be described as ambiguously aggressive.

Following the video, all participants rated two low-level behavior descriptions (“Did Harry intentionally swing his hand down on Tom’s hand?”; “…slap Tom’s hands?”) and two high-level behavior descriptions (“Did Harry intentionally hurt Tom?”; “…make Tom feel uncomfortable?”) using a 1 (Not at all) to 7 (Completely) Likert scale. Participants also rated four possible motives for Harry’s behavior as to whether they were important reasons for his behavior (“He wanted to play the game”; “…win the game”; “…hurt Tom”; “…make Tom cry out in pain”) on a 1 (Most definitely not) to 7 (Most definitely) Likert scale. After participants responded to all of the dependent measures, they were asked to indicate their age and gender and then were thanked for their participation and debriefed.

### Results

#### Data Reduction

As in the previous study we combined the two low-level behavior descriptions (swinging his hands and slapping Tom’s hand) and the two high-level behavior descriptions (hurting Tom; making Tom uncomfortable) into “low-level” and “high-level” behavior measures, respectively. As in Study 1, pretesting on a separate group of participants online (*N* = 37) revealed that people viewed the low-level behavior descriptions to be more concrete (*M* = 3.55 *SD* = 1.60) than the high-level behavior descriptions (*M* = 4.28 *SD* = 1.53), *t*(36) = -2.09, *p* = .04 (Scale 0 = very concrete; 6 very abstract).

Similarly, the individual motive items were combined into composite “positive motive” (wanting to play the game; wanting to win) and “revenge motive” (wanting to hurt Tom; wanting to make Tom cry out) measures. These composite measure were significantly negatively correlated *r*(96) = -.53, *p* < .001. (See S2 Table for descriptive statistics for all individual intentionality and motive items).

#### Manipulation Check

We tested whether the vignette successfully manipulated participant’s perceptions of the target person’s motives. As predicted, participants believed that Harry was more motivated by revenge in the revenge condition (*M* = 3.6 *SD* = 1.1) than in the positive motive condition (*M* = 2.5 *SD* = 1.1), *t*(94) = 5.1, *p* <.001, *d* = 1.0. Similarly, participants believed that Harry was more motivated by positive motives (e.g., just wanting to play and win the game) in the positive motive condition (*M* = 5.8 *SD* = .96), compared to the revenge motive condition (*M* = 5.2 *SD* = .93), *t*(94) = -2.81, *p* = .006, *d* = .64.

#### Perceptions of Intentionality

**Fig 2:**
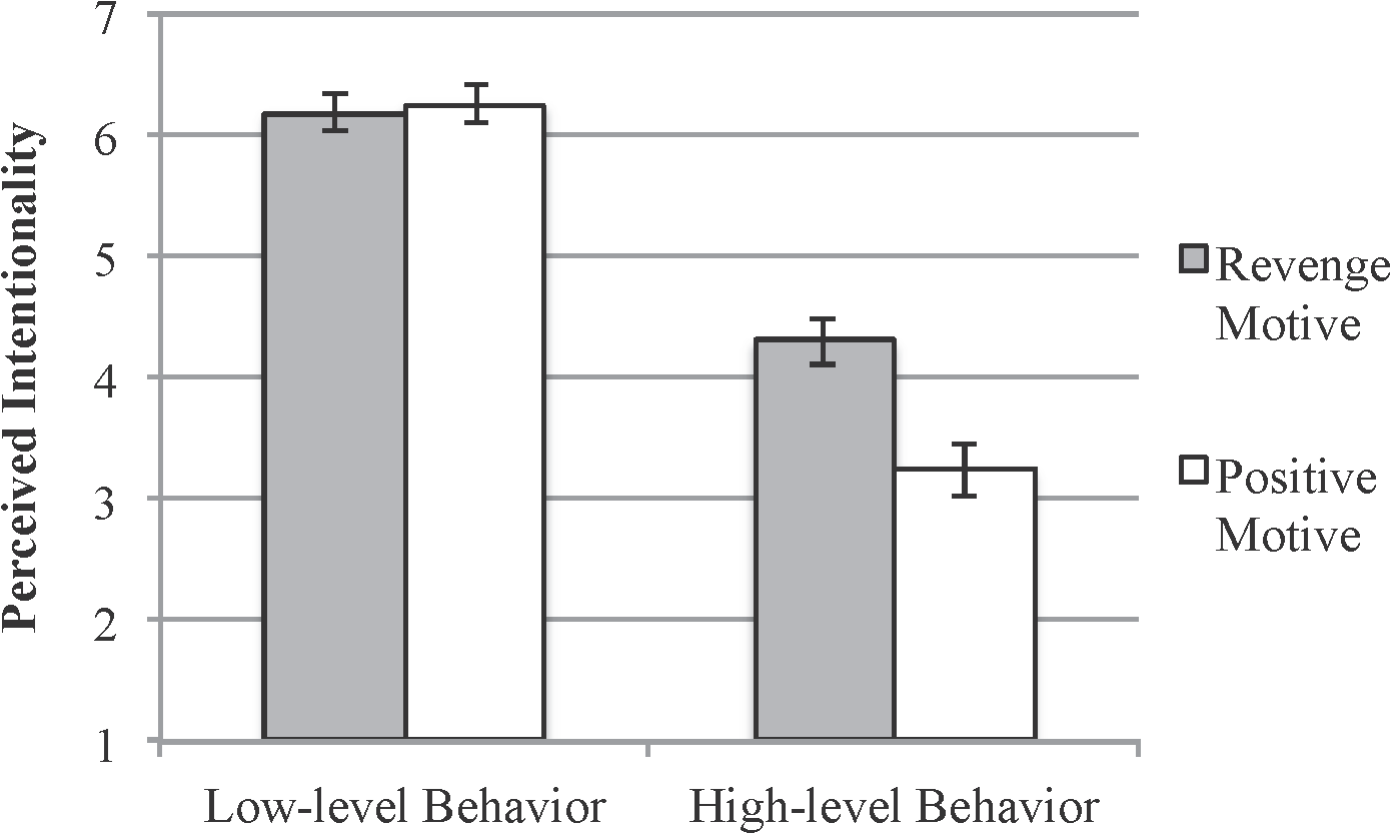
Intentionality judgments for low-level and high-level behavior descriptions as a function of motive information. Error bars = ±1 SE.

Perceptions of intentionality are displayed in Fig. 2. These ratings were subjected to a 2 (Motive: positive vs. revenge) x 2 (Type of Event: low-level vs. high-level behavior description) mixed-model ANOVA. Replicating the results of Study 1, low-level behavior descriptions were rated as more intentional (*M* = 6.22 *SD* = 1.09) than high-level behavior descriptions, (*M* = 3.78 *SD* = 1.53), *F*(1,94) = 180.5, *p* < .001, *d* = 1.8. Also, consistent with previous research on motive-matching (Monroe & Reeder, 2011), there was a significant effect of the agent’s motives on intentionality, *F*(1,94) = 6.91, *p* = .01, *d* = .32. When Harry’s motive (revenge) seemed to match his aggressive, slapping behavior, he was judged as acting more intentionally (*M* = 5.24 *SD* = 0.92) than when his motive (e.g., to win the game) did not match his behavior (*M* = 4.75 *SD* = 0.93).

A critical test of our model is whether an agent’s motives differentially affect perceptions of intentionality for high and low-level behavior descriptions. This interaction was statistically reliable, *F*(1,94) = 9.70, *p* = .002, *d* = .90. Simple effects revealed that the motive manipulation had a significant effect on judgments of intentionality for high-level behavior descriptions. Perceived intentionality for high-level behaviors was greater when the agent was motivated by a revenge motive (*M* = 4.31 *SD* = 1.38) than when the agent had a positive motive, (*M* = 3.24 *SD* = 1.50), *t*(94) = 3.6, *p* <.001. However, for low-level behavior descriptions, judgments of intentionality did not differ between the revenge (*M* = 6.18 *SD* = 1.10) and positive motive (*M* = 6.26 *SD* = 1.10) conditions, (*t*[94] = -.32, *p* >.7).

### Study 2 Discussion

The previous two studies demonstrated a robust effect of social context and mental state information on perceptions of intentionality for high-level behaviors. By contrast, judgments of intentionality for low-level behaviors were largely independent of these sources of information, and remained high across both studies. Thus, consistent with action identification theory [22–23], judgments of intentionality for high and low-level behavior construals appear to be distinct from one another. Judgments of intentionality for low-level behavioral construals remained relatively high [30–32], whereas people used information about social context and motive to disambiguate the meaning of higher-level construals.

One alternative explanation for these findings is that low-level behaviors (e.g., picking up a pen) are relatively proximal to the intent to act (wanting to hold the pen); whereas, high-level behaviors (e.g., helping needy children) are the product of an extended sequence of events (e.g., Helping needy children occurred only after the businessman called his accountant, picked up a pen, and signed a check). Thus, the length of the causal chain linking intentions to high-level goals may dampen judgments of intentionality (perhaps due to the possibility of other intervening causes). We test this possibility in Study 3.

## Study 3

Study 3 manipulated the length of the causal chain of events that lead to the completing a high-level behavior. If the extended causal chain account has merit, perceivers should judge high-level behaviors as less intentional when they result from a long series of causes compared to when they are accomplished immediately. Additionally, we manipulated the agent’s motives, providing another test of the prediction that motives specifically guide judgments of intentionality for high-level behaviors. Finally, we utilized a new scenario in order to further test the generality of our findings.

### Methods

#### Participants

The study was conducted online using Amazon Mechanical Turk (AMT). In total, 327 participants began the experiment; 30 subjects were omitted from the final analysis for not completing the study or failing to follow directions. Participants were paid $0.30 for participating. The remaining 297 subjects had an average age of 32.5 years (*SD* = 12.2). The sample was evenly split between men (*n* = 159) and women (*n* = 133), with five participants declining to indicate their gender.

The sample represented a diverse range of education: 12.8% (*n* = 38) reported finishing high school as their highest level of education; 41.9% (*n* = 124) reported some college experience or attaining a 2-year degree; 33.4% (*n* = 99) attained a 4-year degree; and 11.8% (*n* = 35) reported attaining a Master’s degree or higher. Political attitudes of the sample were slightly liberal, *M* = 3.48 (*SD* = 1.62) on a 1–7 scale (1 = Very liberal; 4 = moderate; 7 = Very conservative).

#### Design, Procedure, and Materials

After viewing a short description of the experiment, participants read the informed consent and indicated their consent to participate in the study by clicking a button labeled “I consent to participate.” After providing informed consent, participants were randomly assigned to one of four conditions. Each condition described an agent named John who caused his uncle’s death by giving him a heart medication.

The between-subjects portion of the design manipulated the length of the causal chain (short or long) that led to the uncle’s death and John’s motives (helpful or harmful). In the *short causal chain condition*, the uncle immediately died from an overdose. By contrast, the *long causal chain condition*, contained three intermediary steps between from taking a heart medication and the uncle’s death: being rushed to emergency surgery; requiring a blood transfusion; and a shortage of blood stocks at the hospital (See S1 Supplementary Materials for vignette text).

After reading the vignette, all participants were asked to rate the intentionality 1 (not at all intentional) to 7 (completely intentional) of two *low-level behavior descriptions* (picking up the bottles of pills and offering the pills to his uncle) and two *high-level behavior descriptions* (killing the uncle and causing his uncle to overdose). Additionally, participants rated the importance of two helpful motives (wanting to help his uncle get better; wanting to take care of his uncle) and two harmful motives (wanting to kill his uncle; wanting his uncle to have a heart attack) for determining John’s actions, using a 1 (most definitely not) to 7 (most definitely) Likert scale. The order of the motive and intentionality judgments was counterbalanced across conditions. After completing the dependent measures, participants provided their demographic information and read the debriefing statement.

### Results

#### Data Reduction

As in the previous study we combined the two low-level behavior descriptions (picking up the pills and offering the pills to his uncle) and the two high-level behavior descriptions (killing the uncle and causing his uncle to overdose) into their respective composite items. Similarly, the individual motive items were combined into composite “harmful motive” (wanting to kill his uncle and wanting his uncle to have a heart attack) and “helpful motive” (wanting to help his uncle get better and wanting to take care of his uncle) measures. These motive measures were significantly negatively correlated *r*(295) = -.94, *p* < .001. (See S1 Supplementary Materials for descriptive statistics for all individual intentionality and motive items).

#### Testing Judgments of Intentionality

**Fig 3:**
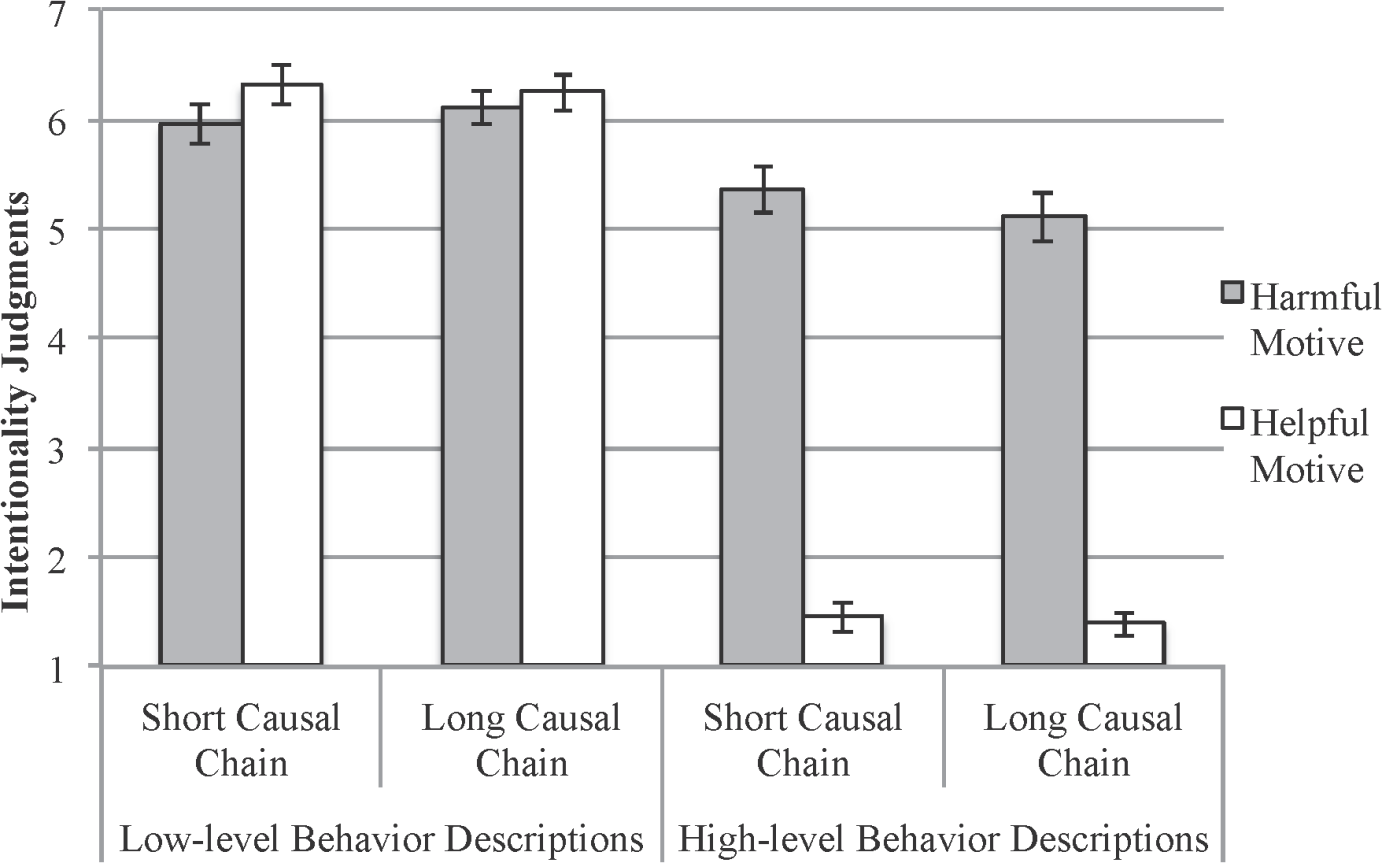
Intentionality judgments for low-level and high-level behavior descriptions as a function of motive and causal chain manipulations. Error bars = ±1 SE.

A 2 (causal chain) x 2 (motive) x 2 (low vs. high-level behavior description) mixed-model ANOVA tested the effects of the causal chain and motive manipulations on judgments of intentionality. We replicated the effects from the previous two studies (see Fig. 3). Overall, low-level behaviors (*M* = 6.15 *SD* = 1.46) were perceived as more intentional than high-level behaviors (*M* = 3.40 *SD* = 2.46), *F*(1, 292) = 593.8, *p* < .001, partial *d* = 1.36. Also, there was a significant effect of the agent’s motives on intentionality, *F*(1, 292) = 180.3, *p* < .001, *d* = 1.56. When the agent had a motive (i.e., he wanted to kill the uncle) that matched the lethal outcome, he was judged as acting intentionally (*M* = 5.63 *SD* = 1.14); but when he had a motive (i.e., wanting to help the uncle get better) that did not match the lethal outcome he was judged as acting unintentionally (*M* = 3.85 *SD* = 1.14).

Finally, we tested the prediction that high and low-level behavior descriptions would be differentially sensitive to motive information. The predicted interaction was significant, *F*(1, 292) = 306.8, *p* < .001, partial η^2^ = .51. The agent’s motives significantly influenced people’s ascriptions of intentionality for high, but not low-level behaviors (Fig. 3). By contrast, the causal chain manipulation did not significantly affect intentionality ascriptions; nor did it interact with the motive manipulation or the behavior description (*Fs* < 1). A post-hoc power analysis showed that we had sufficient power (1-β = .84) to detect an effect of the causal chain manipulation if there was one.

## General Discussion

Perceptions of intentionality critically guide everyday social interactions, though researchers often stress different themes explaining how people infer intentionality. On the one hand, there is evidence that people are biased toward labeling behaviors as intentional [14–15]. On the other hand, research suggests that judgments of intentionality are systematic, building on perceptions of others’ mental states and social context [4, 19–21].

### Working Towards Theoretical Integration

Drawing on action identification theory [22–23], we proposed that judgments of intentionality are not of a single kind. Instead, people parse intentionality into judgments about low-level, concrete behaviors and high-level, abstract behaviors, with information about social context playing different roles at each level. At a low-level of action identification, intentionality judgments are high and relatively insensitive to social context information, consistent with work on the “intentionality bias” [14–16]. Hearing that “Fred slapped Matilda” does not require people to search for information about motive or context to judge intentionality. The verb “slapped” activates the appropriate social scripts [32], and people easily label the action as intentional.

By contrast, at a high-level of action identification, judgments of intentionality are shaped by social context and information about agents’ mental states, consistent with a more complex view of perceived intentionality [4, 20–21]. Deciding whether “Nathan hurt Emma’s feelings” intentionally or accidentally requires knowing something about Nathan’s motives. Did Nathan dislike Emma? Was he trying to tell a joke? Without this contextual information, the social meaning of Nathan’s behavior is unclear.

Going forward, we believe the distinction between high and low-level behaviors will prove useful to researchers in this area. Consider, for example, the controversy surrounding the side-effect effect [4, 9, 12]. In this research, a company CEO was perceived as acting more intentionally when his decision had a side-effect that harmed the environment compared to when the side effect helped the environment. One interpretation of this finding is that the valence of an outcome drives perceptions of intentionality. Accordingly, the "badness of the side-effect itself is what influences people’s intuitions about whether it was intentional" ([42] p. 255). An alternative suggested by our data is that judgments of intentionality are shaped by mental state attributions. In the case where the environment is harmed, the CEO’s motivation toward the environment (as he states “I don’t care at all about harming the environment.”) matches the outcome. Accordingly, the harm is seen as intentional. In the case where the environment is helped, however, that same uncaring motivation (i.e., “I don’t care at all about helping the environment.”) seemingly conflicts with the helpful outcome, leading to reduced judgments of intentionality [4, 20].

Our distinction between judgments for low-level concrete actions and high-level, abstract outcomes mirrors the distinction in developmental psychology between children’s “lean” and “rich” interpretation of behavior [43]. Toddlers as young as six months of age can distinguish between intentional and unintentional behavior [33]; yet, an understanding of others’ desires does not emerge until around two years of age [44], and it is not until 3 or 4 years of age that children acquire an appreciation of belief [48]—both key criteria for adult judgments of intentionality [19]. Some recent research suggests that children may acquire belief concepts much earlier than three years of age. New research places the emergence of false belief understanding within the second year of life [45–47]. If correct, these findings would compress (though leave intact) the timeline for the lean vs. rich concept of intentionality.

Thus lacking key ingredients for conceptually rich judgments, children may initially rely on a lean concept that responds to low-level, physical cues (e.g., body movements, gaze direction, repetition). This concept is then refined throughout development as children acquire an understanding of desire and belief.

This conceptual development may leave adults with two methods for evaluating intentionality: one based on a conceptually lean interpretation of behavior and another based on a conceptually rich interpretation of behavior, with each responding to different types of information—lean responding to motor and verbal cues and rich responding to social and mental state information. The cognitive mechanisms used to make judgments of low-level behaviors may be conceptually simpler and therefore more automatic and less effortful (which would mesh with the intentionality bias account); whereas, the cognitive mechanisms required for judgments of high-level behaviors may be conceptually complex and therefore more effortful. Future work could test this hypothesis by examining judgments of high and low-level behavior descriptions while under cognitive load. Judgments of low-level behavior descriptions should presumably be unaffected by cognitive load; however, judgments of high-level behavior descriptions—when made under cognitive load—should show less reliance on mental states and social information and a heavier reliance on scripts and linguistic cues.

### Possible Limitations and Replies

Despite the strong patterns in our data, several limitations must be acknowledged, and some alternative interpretations remain. One possible limitation is that our findings may result from an inherent difference in the moral valence of low-level and high level behaviors. In our studies, low-level behavior descriptions are morally neutral, while high-level behavior descriptions are morally valenced (positively in Study 1 and negatively in Studies 2 and 3). Thus, the asymmetrical effect of mental state information on intentionality judgments could simply reflect deeper processing of high-level behavior descriptions—where blame and praise are ‘on the line’—relative to low-level behavior descriptions that lack moral valence. If this is correct, it is possible that our results will not replicate in scenarios where *both* high and low-level behavior descriptions lack moral relevance.

An additional study (Study 4, see S2 Supplementary Materials) examined this alternative explanation by replicating the coercion manipulation from Study 1, but with a morally neutral outcome (e.g., changing the color of a widget). Stimuli were adapted from Uttich and Lombrozo [49] who specifically designed their stimuli to be free of moral valance. If the moral valence difference between the high and low-level behavior descriptions explains our effects, then making high-level behavior descriptions morally neutral should eliminate the effects. We found, however, that when participants reasoned about morally neutral, low-level behavior descriptions (e.g., pulling a lever) and morally neutral, high-level behavior descriptions (e.g., changing the color of a widget) our earlier results replicated. Participants used information about social context and motive to guide their judgments about high-level behavior descriptions, while judgments of low-level behavior descriptions remained unaffected by context (S2 Supplementary Materials).

A second additional study (Study 5, see S3 Supplementary Materials) addressed a separate alternative interpretation regarding our response scales for perceived intentionality. It may be that low-level behaviors are viewed as dichotomous—being either accidental or intentional; whereas, high-level behaviors are seen as arising from more nuanced aspects of intentionality. By asking about intentionality, rather than whether a behavior was accidental, it is possible that our response scales were biased towards assessing impressions of high-level behavior descriptions. To examine this possibility we replicated the helpful and harmful conditions of Study 3 (short-causal chain only), but instead of asking about intentionality, the scale asked if the high and low-level behaviors were completed *accidentally* (e.g., Did John accidentally pick up a bottle of pills?; 1 = Not at all, 7 Completely). The altered response scales did not affect the obtained results in any significant way, and we replicated our previous findings (S3 Supplementary Materials).

### Future Directions and Conclusions

Although we did not find any evidence that a longer casual chain of events influenced perceptions of intentionality (Study 3), this factor may play a role under other circumstances. For example, if a long series of low-level behaviors is necessary to complete a high-level behavior—such as having a novel accepted for publication—perceivers may be inclined to attribute *higher* intentionality. The sustained effort (and long chain of events) needed to get published might imply high levels of planning and intelligence, which are positively associated with attributions of agency and intentionality [27, 50–51].

Finally, our data contrast to some degree with current hierarchical theories of action planning [52], which argue that low-level behaviors are always determined by high-level behaviors and goals. For example, the goal of having a romantic anniversary dinner determines the necessary subsequent low-level behaviors and their meaning (e.g., calling for a reservation, driving to the restaurant, selecting a nice wine). Such a hierarchical model would predict, in contrast with our findings, that perceptions of intentionality for low-level actions (e.g. selecting a nice wine) should be determined by higher-level goals (e.g. having a romantic dinner). We propose that the apparent conflict between the action planning approach and our own findings may relate to differences in behavioral planning by actors versus the explanations offered by observers of those actors.

When planning their own actions, agents must first decide on their desired ultimate end in order to determine the specific steps they must take to accomplish it. By contrast, when explaining the behavior of others, observers have imperfect knowledge of the agent’s mind. Observers must decode the simpler, concrete aspects of behavior and scaffold up to explain an agent’s abstract goals. In the process, our data suggest that social context and the agent’s motives are useful for interpreting and explaining higher-level behaviors. The present studies, however, only speak to the latter inference problem (explaining someone else’s behavior). Future studies may be able to reconcile our account with hierarchical theories of action planning by constructing a self vs. other contrast in the research.

The present studies are a first step toward exploring differences in the way low and high-level behavioral descriptions are perceived. We hope that future work expands on these findings to examine possible boundary conditions. It is possible that social and mental state information may play a more prominent role in shaping people’s judgments about canonically unintentional or ambiguous behaviors. In addition, all of our studies included manipulations of either social context or motive. Future studies might examine judgments of intentionality for simple descriptions of actions and their associated outcomes, without including additional information. By doing so, researchers could examine a wider variety of behavioral events and more easily test for moderating influences.

## Supporting Information

S1 Data Studies 1–5 Raw data. (ZIP)

S1 Fig Mean judgments of intentionality for morally neutral low and high-level behaviors by coercion condition. Error bars = ±1 SE.
(TIFF)

S2 Fig Judgments of whether an agent’s low and high-level behaviors were accidental (1 = not at all accidental; 7 = completely accidental) across a between subjects manipulation of motive (harm vs. help). Error bars = ±1 SE.
(TIFF)

S1 Supplementary Materials Study 3: Stimuli and descriptive statistics for individual intentionality and motive dependent variables. (DOCX)

S2 Supplementary Materials Study 4: Amoral action and outcome study. (DOCX)

S3 Supplementary Materials Study 5: Replication of Study 3 with accidental dependent variables. (DOCX)

S1 Table Study 1: Descriptive statistics for individual intentionality and motive dependent variables. (DOCX)

S2 Table Study 2: Descriptive statistics for individual intentionality and motive dependent variables. (DOCX)
