# Supplementary figures and images for: Perceptions of Intentionality for Goal-Related Action: Behavioral Description Matters
Source: PLoS One. 2015 Mar 17;10(3):e0119841. doi: 10.1371/journal.pone.0119841 (PMC4362945; doi:10.1371/journal.pone.0119841)

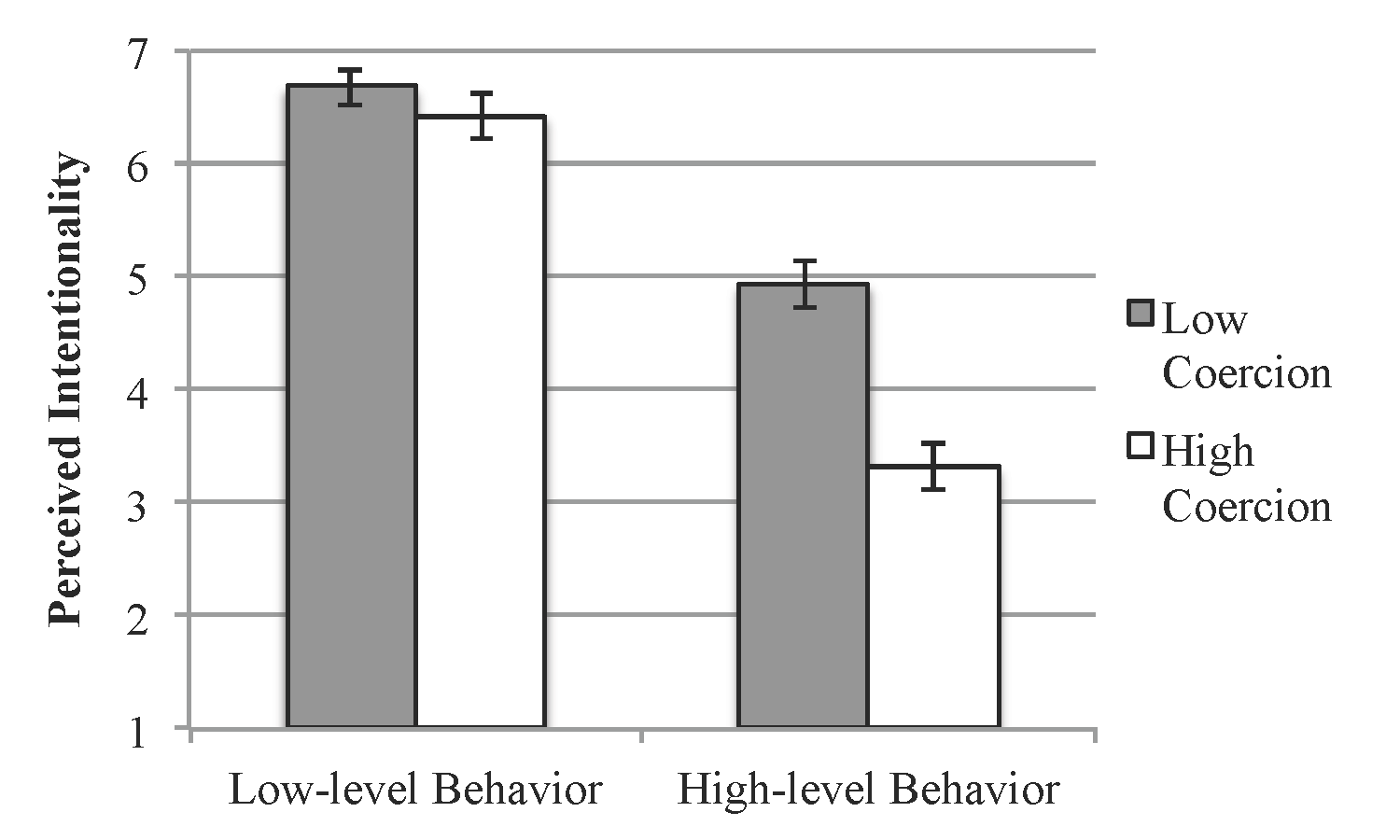

Supplement: S1 Fig — Error bars = ±1 SE. (TIFF) [file pone.0119841.s002.tiff]

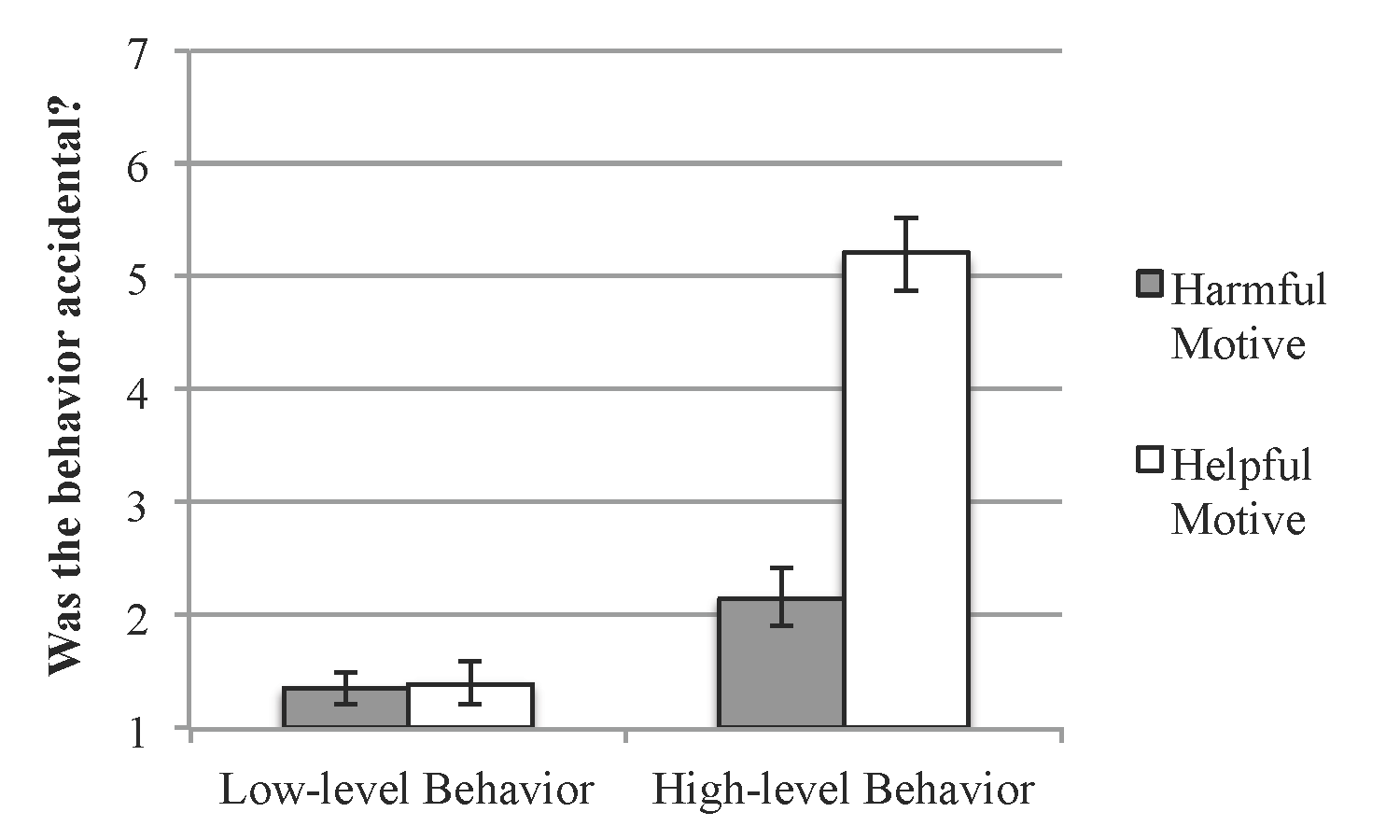

Supplement: S2 Fig — Error bars = ±1 SE. (TIFF) [file pone.0119841.s003.tiff]
