# Supplementary material for: Perceptions of Intentionality for Goal-Related Action: Behavioral Description Matters
Source: PLoS One. 2015 Mar 17;10(3):e0119841. doi: 10.1371/journal.pone.0119841 (PMC4362945; doi:10.1371/journal.pone.0119841)
Supplement: S1 Supplementary Materials — (DOCX) [file pone.0119841.s004.docx]

# S1 Supplementary Materials

## Study 3 Stimuli (motive manipulation in brackets)

### Short causal chain:

“John's uncle was recently admitted to the hospital with chest pains. John [hated/was very fond of] his uncle and wanted to [kill him/help him get better]. One day when John visited his uncle in the hospital, the on-call nurse had to leave to attend to another patient. She asked John to give his uncle his heart medication and then left the room. John picked up the medication, measured out several pills, and gave them to his uncle. A few minutes after swallowing the pills John's uncle had a heart attack due to an overdose of medication, which immediately killed him. John was [happy/devastated] about his uncle's death.”

### Long causal chain:

“John's uncle was recently admitted to the hospital with chest pains. John [hated/was very fond of] his uncle and wanted to [kill him/help him get better]. One day when John visited his uncle in the hospital, the on-call nurse had to leave to attend to another patient. She asked John to give his uncle his heart medication and then left the room. John picked up the medication, measured out several pills, and gave them to his uncle. A few minutes after swallowing the pills John's uncle had a heart attack due to an overdose of medication. He was sent into emergency surgery. During the long surgery John's uncle lost a lot of blood, but the doctor could not perform a blood transfusion because the hospital recently cut its stocks of blood to save money. Because of this John's uncle lost too much blood and died. John was [happy/devastated] about his uncle's death.”

S3 Table.

*Study 3: Descriptive statistics for individual intentionality and motive dependent variables*

|  |  | Motive Condition | | | |
| --- | --- | --- | --- | --- | --- |
|  |  | Harmful Motive | | Helpful Motive | |
|  |  | *M* | *SD* | *M* | *SD* |
| **Low-level Behavior Descriptions** | |  |  |  |  |
|  | Pick up the bottle of pills | 5.83 | 1.86 | 6.24 | 1.63 |
|  | Offer the pills to his uncle | 6.24 | 1.49 | 6.32 | 1.44 |
| **High-level Behavior Descriptions** | |  |  |  |  |
|  | Kill his uncle | 5.17 | 1.99 | 1.36 | 1.11 |
|  | Give his uncle an overdose | 5.32 | 1.95 | 1.48 | 1.28 |
| **Motive Items** | |  |  |  |  |
|  | Wanted to help his uncle get better | 2.06 | 1.35 | 6.36 | 1.06 |
|  | Wanted to take care of his uncle | 2.01 | 1.37 | 6.35 | 1.03 |
|  | Wanted to his uncle to have a heart attack | 4.96 | 1.62 | 1.44 | 0.99 |
|  | Wanted to kill his uncle | 5.71 | 1.55 | 1.39 | 0.96 |
