# Supplementary material for: Perceptions of Intentionality for Goal-Related Action: Behavioral Description Matters
Source: PLoS One. 2015 Mar 17;10(3):e0119841. doi: 10.1371/journal.pone.0119841 (PMC4362945; doi:10.1371/journal.pone.0119841)
Supplement: S2 Supplementary Materials — (DOCX) [file pone.0119841.s005.docx]

# S2 Supplementary Materials

## Study 4: Amoral action and outcome study

### Participants and Procedure

Eighty participants were recruited via Amazon Mechanical Turk. Participants were randomly assigned to read either a low coercion or high coercion story. Both stories described a man who changed the color the widgets his company produced. In the low coercion condition Bob decided to change the widgets’ color on his own; in the high coercion condition Bob decided to change the widgets’ color after being threatened. We omitted six participants from the analyses for failing the attention check question (“In the story, the usual procedure at Gizmo industries is to make widgets what color?”) (final *n* = 74).

After reading the story, participants responded to three low-level behavior description questions (“Did Bob intentionally open the color control console?”; “…turn the "red" and "blue" knobs four clicks each?”; “…pull the activation lever down?”) and three high-level behavior description questions (“Did Bob intentionally change what the factory produced?”; “…amuse Gizmo's customers?”; “…surprise Gizmo's customers?”) using a 1 (not at all) – 7 (completely) Likert scale. The order of the low-level and high-level behavior description questions were counterbalanced across participants.

Participants also responded to four motive questions using a 1 (most definitely not) – 7 (most definitely yes) Likert scale. Two questions measured the motive to change the widgets color (“He wanted to brighten up the color of his company's widgets.”; “He wanted to change the widgets' color to something less boring.”), and two questions measured the motive to protect himself (“He wanted to save his own life.”; “He wanted to protect himself.”).

### Stimuli (Coercion manipulation in brackets)

A man named Bob is the night foreman at the Gizmo industries factory. The factory that Bob works at produces widgets for various machines. The usual procedure at Gizmo industries is to make widgets that are a dark color. Specifically, the usual procedure is to make widgets that are colored dark blue.

[*Low coercion*: One day while sitting at home, Bob thought to himself “those dark blue widgets are so boring; we should lighten them to something like bright red.]

[*High coercion*: One day a man broke into Bob’s house and tied him up. The man, clearly dangerous and deranged, told Bob: “those dark blue widgets are so boring; you should lighten them to something like bright red.” The man told Bob, that unless Bob changed the widget color to bright red, he would return in one week and kill him. The man said that if Bob went to the police, he would still find a way to kill him. The man then untied Bob and left his home.]

The next night Bob went to the factory controls. He opened the main color control console. Bob turned the “blue” knob down 4 clicks, and he turned the “red” knob up 4 clicks. Bob then pulled the main activation lever down, activating the widget machine. The machine did its work and produced bright red widgets.

The new bright red widgets surprised Gizmo’s customers, who were a little confused, but slightly also slightly amused by the new color of the widgets.

### Results

We replicated both of our primary findings from the previous Studies. Participants rated low-level behavior descriptions as more intentional than high-level behavior descriptions, *F*(1,72) = 193.8, *p* < .001, *d* = 1.87 (See Figure 4). Also, the predicted coercion by event interaction was significant, *F*(1,72) = 15.2, *p* < .001, *d* = 1.21. Coercion lowered participants’ judgments of intentionality for high-level behavior descriptions, while judgments for low-level behavior descriptions were largely unaffected. Finally, we replicated the significant main effect of coercion on perceptions of intentionality, *F*(1,72) = 66.8, *p* < .001, *d* = 1.06. Participants rated the agent’s behavior as less intentional when he was coerced into changing the widgets’ color compared to when he decided for himself.

**Fig. S1. Mean judgments of intentionality for morally neutral low and high-level behaviors by coercion condition. Error bars = ±1 SE**
