# Supplementary material for: Perceptions of Intentionality for Goal-Related Action: Behavioral Description Matters
Source: PLoS One. 2015 Mar 17;10(3):e0119841. doi: 10.1371/journal.pone.0119841 (PMC4362945; doi:10.1371/journal.pone.0119841)
Supplement: S3 Supplementary Materials — (DOCX) [file pone.0119841.s006.docx]

# S3 Supplementary Materials

## Study 5: Replication of Study 3 with accidental DV’s

### Participants and Procedure

Eighty participants were recruited via Amazon Mechanical Turk. Participants were randomly assigned to read either the “help” or the “hurt” version of the “short causal chain” story from Study 3. We omitted five participants from the analyses. Two participants did not complete any of the dependent measures, and three participants failed the attention check question (“In the story, who gave the uncle the heart medication?”) (final *n* = 75).

After reading the story, participants were asked to make judgments about whether various behaviors performed by John’s were *accidental*. We used the same dependent measures as Study 3, but we replaced the word “intentionally” with “accidentally” in all of the questions: Two questions measured perceptions of high-level behavior descriptions (“Did John accidentally kill his uncle?”; “Did John accidentally give his uncle an overdose of medication?”), and two questions measured perceptions of low-level behavior descriptions (“Did John accidentally pick up the bottle of pills?” “Did John accidentally offer the pills to his uncle?”). Each question used a 1 (not at all) – 7 (completely) Likert scale. Low and high-level behavior descriptions were blocked together, and the order of the blocks was counterbalanced across participants.

Participants also responded to four motive questions using a 1 (most definitely not) – 7 (most definitely yes) Likert scale. Two questions assessed helpful motives (“He wanted to help his uncle get better.”; “He wanted to take care of his uncle.”), and two questions assess harmful motives (“He wanted to kill his uncle.”; “He wanted his uncle to have a heart attack.”).

### Results

Exchanging the word “intentionally” for the word “accidentally” in our dependent measures lead to a set of findings that mirrored the previous studies. Participants rated high-level behavior descriptions as more accidental than low-level behavior descriptions, *F*(1,73) = 103.2 *p* < .001, *d* = 1.23 (see Figure 5). We also found the predicted coercion by event interaction, *F*(1,73) = 43.9 *p* < .001, *d* = 1.57. Participants generally rejected the possibility that the agent’s low-level behaviors were accidental, but judgments of high-level behaviors depended on the agent’s motives. Lastly, the effect of motive on participants’ intentionality judgments also replicated, *F*(1,73) = 39.5 *p* < .001, *d* = 1.45. Participants were more likely to judge behavior as accidental when the agent’s motive did not match with his behavior than when behavior and motives matched.

**Fig. S2. Judgments of whether an agent’s low and high-level behaviors were accidental (1 = not at all accidental; 7 = completely accidental) across a between subjects manipulation of motive (harm vs. help). Error bars = ±1 SE**
