# Supplementary material for: Perceptions of Intentionality for Goal-Related Action: Behavioral Description Matters
Source: PLoS One. 2015 Mar 17;10(3):e0119841. doi: 10.1371/journal.pone.0119841 (PMC4362945; doi:10.1371/journal.pone.0119841)
Supplement: S1 Table — (DOCX) [file pone.0119841.s007.docx]

S1 Table.

*Study 1: Descriptive statistics for individual intentionality and motive dependent variables*

|  |  | Level of Coercion | | | |
| --- | --- | --- | --- | --- | --- |
|  |  | Low coercion | | High Coercion | |
|  |  | *M* | *SD* | *M* | *SD* |
| **Low-level Behavior Descriptions** | |  |  |  |  |
|  | Call his accountant | 6.46 | 0.79 | 4.96 | 2.36 |
|  | Meet with school officials | 5.96 | 1.48 | 4.79 | 2.41 |
|  | Write a $10 million check | 6.11 | 1.42 | 4.54 | 2.32 |
| **High-level Behavior Descriptions** | |  |  |  |  |
|  | Help the children | 5.61 | 1.40 | 2.18 | 1.54 |
|  | Give money to a charitable cause | 5.61 | 1.23 | 3.04 | 2.17 |
|  | Do a good deed | 5.82 | 1.19 | 2.11 | 1.69 |
| **Motive Ctems** | |  |  |  |  |
|  | Wanted to stay alive | 2.39 | 1.29 | 6.86 | 0.45 |
|  | Wanted to protect himself | 3.57 | 1.60 | 6.71 | 0.66 |
|  | Wanted to be altruistic | 4.79 | 1.34 | 2.85 | 1.70 |
|  | Wanted to help underprivileged kids | 5.21 | 1.32 | 2.07 | 1.15 |
