# Supplementary material for: Perceptions of Intentionality for Goal-Related Action: Behavioral Description Matters
Source: PLoS One. 2015 Mar 17;10(3):e0119841. doi: 10.1371/journal.pone.0119841 (PMC4362945; doi:10.1371/journal.pone.0119841)
Supplement: S2 Table — (DOCX) [file pone.0119841.s008.docx]

S2 Table.

*Study 2: Descriptive statistics for individual intentionality and motive dependent variables*

|  |  | Motive Condition | | | |
| --- | --- | --- | --- | --- | --- |
|  |  | Positive Motive | | Revenge Motive | |
|  |  | *M* | *SD* | *M* | *SD* |
| **Low-level Behavior Descriptions** | |  |  |  |  |
|  | Swing his hand down | 6.14 | 1.17 | 6.28 | 1.08 |
|  | Slap Tom's hand | 6.22 | 1.18 | 6.23 | 1.22 |
| **High-level Behavior Descriptions** | |  |  |  |  |
|  | Made Tom feel uncomfortable | 4.22 | 1.54 | 3.11 | 1.55 |
|  | Hurt Tom | 4.39 | 1.51 | 3.38 | 1.71 |
| **Motive Items** | |  |  |  |  |
|  | Wanted to play the game | 4.63 | 1.39 | 5.60 | 1.23 |
|  | Wanted to win the game | 5.90 | 0.94 | 6.02 | 1.03 |
|  | Wanted to hurt Tom | 4.16 | 1.21 | 2.83 | 1.32 |
|  | Wanted to make Tom cry out in pain | 3.12 | 1.18 | 2.15 | 1.08 |
